# Medical Books

**Published:** 1847-02

**Authors:** 


ARTICLE XV.

### MEDICAL BOOKS.

We are highly gratified to see our book merchants bringing on large and well selected assortments of medical books. Almost any medical work that the physician or student may require can be obtained in Chicago on as good terms as any where in the west. This is a great convenience to the medical public, and we hope that our friends may meet with an ample reward for their enterprise from rapid sales.

While on this subject we would drop a hint to our brethren through the country, that many of them have entirely too meagre a show of medical library, and occasionally we meet with one with scarcely a single medical book. This is all wrong. It is impossible for the practitioner, let him be ever so gifted, to maintain his ground, so far as science is concerned, unless he is a reader of medical books; to say nothing in reference to keeping pace with the great and important improvements that are yearly being made in the several departments of the science.

A friend of ours, a few years ago, said he had adopted as a rule to set apart annually of the proceeds of his practice fifty dollars, to be invested in books. The principle is a good one; and if each practitioner in the country would annually set apart the sum that he should determine before hand to be consistent with his wants and his ability, even if small, and invest it in the most approved works on the different branches of the science, and read them, it would in a few years ornament his office with a well selected library, and, what is more important, his head with a knowledge of the resources of the profession.

The time is coming when the profession in the west will assume a higher and a more dignified stand. We think the time not far distant. The signs look better; and amongst those that are decidedly encouraging are the increased number of students and physicians who attend upon the teachings of the medical schools, and the increase in the sale of medical books. E.

